# Latent Inhibition as a Biological Basis of Creative Capacity in Individuals Aged Nine to 12

**DOI:** 10.3389/fpsyg.2021.650541

**Published:** 2021-03-29

**Authors:** Antonio José Lorca Garrido, Olivia López-Martínez, María Isabel de Vicente-Yagüe Jara

**Affiliations:** ^1^Department of Evolutionary and Educational Psychology, University of Murcia, Murcia, Spain; ^2^Department of Didactics of Language and Literature, University of Murcia, Murcia, Spain

**Keywords:** creativity, selective attention, latent inhibition, intelligence, primary education

## Abstract

This study focuses on latent inhibition, a mechanism behind selective attention, as the biological basis of creativity in schoolchildren. The main objective of this study is to know if low levels of attention positively affect the levels of creativity manifested in students between the ages of nine and 12. The design of this study is non-experimental with an explanatory-correlational cross-sectional quantitative approach. In order to achieve the objective suggested, several education centers located in Murcia were selected, in which 476 students took part in a creativity test (PIC-N), an attention test (D2), and another test about intelligence depending on the educational level (BADYG/E2r or BADYG/E3r). The results obtained showed that selective attention was negatively correlated with graphic creativity, understanding that behind it lies the latent inhibition, and that when certain levels of intelligence are present, this negative correlation increases. In this way, the simultaneous existence of creative and inattentive subjects is demonstrated.

## Introduction

Today's society is fully aware that creativity is one of those qualities most valued in humans. In all areas in which knowledge is applied, including work, an innovative individual has a place, since their divergent thinking pushes them to leave their comfort zone, allowing those individuals to live out of it. Schools should be at the forefront of promoting creative thinking. Authors like Chávez et al. (2020) state that in school, children face situations that they should address and solve in different ways, helping them understand that ideas different than their own are still valuable. Even after this, in a majority of schools, creativity is not something that is given proper attention (Pérez, 2018). It is even necessary to mention that limits are imposed on divergent thinking, with creative thinking being seen as a bad habit (Sternberg, 2007).

Taha et al. (2015) defined creativity as the original way in which individuals face challenges in their daily lives. Creative thinking is noted in different ways and can be developed (López and Martín, 2010). Glaveanu et al. (2019) state that creativity is a human quality that can give meaning to our lives. This capacity is a quality that shows up in similar ways in men and women alike, and gender is not a differentiated factor (Harris, 2004; Espinosa, 2005; Elisondo and Donolo, 2011; Abraham et al., 2014; Soisa, 2015).

The current trend in creative thinking is toward specific domain creativity due to correlational studies reporting low relationships between divergent thinking and expert ratings of creative performance in different domains (Artola et al., 2010; Hernández, 2017). Kaufman and Baer (2004) proposed three domains of creativity: verbal (personal problem solving, communication, and writing); graphic (bodily creativity, in art and printmaking); and scientific (creativity in mathematics and science). Previous works, like those from Sternberg and Lubart (1997), support this idea, stating that, as an individual can perform higher in some areas than others, it is possible that this is due to being creative in one area and not in the other.

Artola et al. (2010) use the sum of verbal or narrative creativity together with graphic creativity to arrive at the idea of general creativity; narrative creativity is the divergent idea in the execution of verbal tasks, while graphic creativity is implicit in the execution of nonverbal tasks, such as drawing. Verbal creativity is the linguistic act that activates not only creative thinking but also a process of written reflection in which language ceases to be conventional to give way to a superior discourse where metaphors, originality, and imagination shape the narrative without losing textual harmony (Sandoval, 2016). On the other hand, graphic creativity is defined as that which develops innovative and effective ideas around any graphic process, especially in drawing (Torrance, 1977).

Carson et al. (2003), psychologists and investigators from Toronto and Harvard University, identified low latent inhibition as one of the biological foundations of creativity, stating that the creative people's brains are in contact with more of the environmental stimulus than non-creative ones, since the process of information does not stop. Important to mention is the fact that they noted the requirement of a high intelligence, to allow a low latent inhibition, not associated with schizophrenia or to a psychosis. Considering this, the research problem that needs to be addressed is whether easily distracted individuals, due to a latent attenuated inhibition, are more creative? Is it necessary to possess a higher intelligence to see this association?

The answer to the inclusion of intelligence in this work can be found in a deficit in the attention element, usually associated with a pathology that can become a creativity advantage in the absence of other creativity strengths, like a high intellectual quotient (Carson et al., 2003). Intelligence is a set of problem-solving abilities (Gardner, 2016). Regarding gender differences in levels of creativity, at early ages they are small or non-existent and it is from adolescence onwards, and especially in adulthood, that these differences become more noticeable (Feingold, 1993). In the school period, Lynn and Irwing (2004) found that there are no significant gender differences between the ages of six and 14.

Attention is one of the superior brain mental functions, allowing one to properly organize information obtained from displaced senses, focalization, or due to irrelevant stimuli inhibition (Hernández, 2017). Based on the inhibition of irrelevant stimuli, surges the concept of latent inhibition which Kaufman (2009) affirmed that it is the capacity to maintain the excess of information, understood like irrelevant stimuli, outside our cognition. Henceforth we will assume that, behind selective attention, we can find the mechanism known as latent inhibition that narrows attention and focus, and limits information influx (Hasher et al., 2007). The absence of a stimuli filter is a disadvantage when an individual tries to concentrate on the achievement of tasks that require a high level of attention, since a wide array of stimuli will have access to the subject's mind (González, 2017).

Latent inhibition will allow a higher number of elements to combine, although not all individuals will notice this due to the distance among the different parts, allowing innovative ideas and, in turn, creative thinking (Carson, 2010). Important to note is the fact that innovative thinking refers to new ideas from association, useful to a specific situation, and the distances among elements makes a more creative process (Mednick, 1962).

Mednick (1962) and Campbell (1960) propose that individual differences in attention focus and concentration are the cause of creativity differences. Most of the investigations reviewed use, Eysenck theory (1995) as a starting point in which low latent inhibition influences the level of cognitive inhibition, present in creativity, that combined with sociocultural variable, cognitive variable (IQ), and motivation, allows for creative thinking. The difference between an individual with a psychosis and an innovative individual, when faced with low latent inhibition, is based on the cognitive variables (Carson et al., 2003). Among the population with Attention Deficit Hyperactivity Disorder (ADHD), it has been proven that the inefficiency of the latent inhibition mechanism promotes innovation (Pritchard et al., 2006; González, 2017).

Mendelsohn and Griswold (1966) defend the idea that creative individuals are more likely to use irrelevant stimuli, incidentally or randomly present. This translates into difficulties with cognitive information selection, since they will not be able to differentiate between important noise stimuli and distractors (Martínez, 2001). Although it would be beneficial when completing innovative tasks, it would be a problem when completing other tasks (Rawlings, 1985; Eysenck, 1995).

Current works about low latent inhibition reinforce the idea of problem-finding as the source of problem-solving (Runco, 1994). When more information enters, the higher the probability is of a combination, which will then generate new ideas. In summary, the amount of information that an individual can manage is directly linked to the creation of an original product or the solution to problems. This is due to cognitive irrelevant stimuli remaining active, allowing a higher level of creativity in the individual, different from less innovative individuals, who show a higher level of focus and attention (González, 2017).

Regarding sex, most works support that there are no significant differences based on gender (Fernández-Castillo and Gutiérrez, 2009; Brickenkamp, 2012; Soisa, 2015). Regarding age, sustained attention increases, due to the use of selective strategies, starting at age 10 (Orjales and Polaino, 1992).

The main goal of this work is to know if low attention levels positively influence creativity levels on students aged nine to 12 years of age. The hypothesis was that students with attenuated latent inhibition and above average intelligence will have higher creativity scores. Specific objectives were:

- To know the levels of creativity, attention, and intelligence in students 9–12 years of age.
- To determine if there are creativity, attention, and intelligence differences based on gender.
- To compare creativity, attention, and intelligence based on educational level and to check if there are any significant differences.

## Materials and Methods

### Participants

In this non-experimental quantitative, cross sectional, correlational study (Ato et al., 2013), a total of 476 students, ages nine to 12, from four elementary schools, from the region of Murcia, Spain, participated. These are public schools. The families of the students in these schools have a low economic level and a low socio-cultural level. Regarding grade level, 152 fourth grade students (31.93%), 164 fifth grade students (34.46%), and 160 sixth grade students (33.61%) participated. By gender, 248 males (52.10%), and 228 females (47.90%) participated.

The participants' selection method was non-probability sampling, known as consecutive, engaging only students belonging to the last phase of Elementary education (Valdivia, 2018). Consecutive sampling is comprehensive since all children in the classrooms of the aforementioned grade levels participated.

### Instruments

Four different tests were used to collect information regarding the different variables of this research project.

**“Prueba de Imaginacion Creativa para niños (PIC-N)” (Creative Imagination Test for Children)** (Artola et al., 2010). This test (α = 0.84) is designed to measure the creativity of children in two specific domains: narrative creativity and graphic creativity. Age range is eight to 12. PIC-N comprises four tasks. The first three assess verbal (narrative) creativity, and the last one graphic creativity. In game one, children have 10 min to write down everything that is happening in a scene from an image of a child opening a chest. In game two, subjects have 7 min to identify all possible uses of a rubber tube. In game three, children have 10 min to describe what would happen if all squirrels turned into dinosaurs. In game four, subjects have 10 min to draw four pictures from given strokes and assign a title to each of the drawings.

**D2 Test of Attention** (Brickenkamp, 2012). This test (α = 0.60) assesses selective and sustained attention in individuals engaged in a task in which irrelevant stimuli has to be ignored, fast and accurately. It is made up of 14 lines, 47 characters each, for a total of 658 characters. The task is to cross out any letter “d” with two marks.

**“Bateria de Aptitudes Diferenciales y Generales E2 renovado (BADYG/E2r)” (Differential and General Skills Battery BADYG/E2r)** (Yuste, 2011). This test (α = 0.79) measures cognitive abilities using three elements, verbal, numeric, and spatial, applied to third and fourth grade Elementary students. This instrument is comprised of six tasks (two for each factor), for a total of 24 items each. The six tests are: analog relations, number problems, logic matrices, sentence completion, numerical calculation, and rotated figures.

**“Bateria de Aptitudes Diferenciales y Generales E3 renovado (BADYG/E3r)” (Differential and General Skills Battery BADYG/E3r)** (Yuste et al., 2011). This test (α = 0.88) measures cognitive abilities (IQ) taking into account three factors, verbal, numeric, and spatial, applied to fifth and sixth grade Elementary students. It is composed of six tasks (two for each factor), for a total of 32 elements each. The six tests are: verbal analogies, number series, logic matrices, sentence completion, number problems, and figure matching.

### Procedure

First of all, school approval was sought and parental permission was provided for their children to participate in the study. Approval obtained, the subjects completed the tasks individually using paper and pencil. Formal instruments were administered by grade level, BADYG/E2r for fourth grade, BADYG/E3r for fifth and sixth grades, in an approximate administration time of 1 h and 15 min for each class. The following day and to avoid students' fatigue, the reminder of the creativity instruments (PIC-N) and attention (D2) were administered over a period of about 40 min and 5 min, respectively, with each class.

### Statistical Analysis

Statistical analysis was completed using the program IBM SPSS Statistics (version 24). A data matrix was used to analyze data regarding the proposed objectives. First, statistical evidence was used to address the variable level in the sample. To address possible differences based on the participants' gender, for variables that fit parametric requirements, the independent samples *t*-test and Mann-Whitney *U*-test, for non-independent samples, were used.

Also, and to address possible differences taking into account the educational level of the participants, a statistical variable analysis using One-way ANOVA was completed. With variables that did not meet parametric requirements, the Kruskal-Wallis *H*-test was used. Finally, a correlational analysis with the selected variables was completed using Pearson's correlation.

## Results

Three types of creativity were analyzed in connection with the first objective: two of them measure as creativity as a specific value, while the third one is general. First, narrative creativity (M = 57.49; SD = 24.65) is highly correlated to graphic creativity (M = 12.87; SD = 4.09), both being heterogeneous groups. General creativity (M = 70.37; SD = 26.02) is the addition of the other two, thus higher levels are reached in a group.

Two different kinds of measurement are taken into account: the total effectiveness of the test (TOT) and the concentration index (CON). Total effectiveness of the test (M = 267.96; SD = 70.45) is higher than the concentration index (M = 89.49; SD = 38.51), and both are heterogeneous groups. Regarding cognition, scores come from the Intelligence Quotient (M = 92.68; SD = 16.61), which is also a heterogeneous group.

Before attempting an inferential analysis, it is necessary to probe that these variable samples follow a regular distribution pattern that supports the use of the proper parametric and non-parametric instruments. To accomplish that goal, the Kolmogorov-Smirnov method was applied to each of them. If *p* > 0.5 is accepted as a null hypothesis, homogeneity exists between variables distribution and regular distribution. Thus, narrative creativity (*p* > 0.5), graphic creativity (*p* > 0.5), general creativity (*p* > 0.5), total test effectiveness (*p* > 0.5), and cognition (*p* > 0.5) are distributed following a regular pattern. If *p* < 5, the hypothesis of this research project, stating that there are differences, will be accepted. The concentration index (*p* < 0.5) is not distributed in a regular pattern.

Since the second specific goal was to establish differences based on gender, independent samples *t*-tests were used (Table 1). The *T*-test shows that there are no significant differences based on gender among participants regarding narrative creativity (*t* = −0.636, *p* > 0.05), graphic creativity (*t* = 0.054, *p* > 0.05), general creativity (*t* = −0.595, *p* > 0.05), total effectiveness of the test (*t* = −0.471, *p* > 0.05), or intelligence (*t* = 0.693, *p* > 0.05). As previously mentioned, the concentration index does not follow a regular pattern. For this reason, a non-parametric approach, the Mann-Whitney *U*-test, was used for the analysis of the independent samples *t*-test. *T*-test results (*Z* = −1.059, *p* > 0.05) shows that there are no significant differences based on gender in the concentration index.

**Table 1:** Gender differences in the levels of the variables.

**Variables**	***t***	***p***	**Average difference**	**Standard error difference**
Narrative creativity	−0.636	0.526	−2.890	4.548
Graphic creativity	0.054	0.957	0.042	0.773
General creativity	−0.595	0.553	−2.8489	4.7903
Total test effectiveness	−0.471	0.639	−6.108	12.971
Intelligence	0.693	0.490	2.212	3.072

The third specific goal was to compare creativity levels, attention, and intelligence, based on educational level, to observe any significant differences. This task was addressed using One-way ANOVA inferential analysis, as recorded in Table 2.

**Table 2:** Differences according to educational level in the levels of the variables.

**Variables**	**Grades**	***n***	**M**	**SD**	***F***	***p***
Narrative creativity	Fourth	38	52.93	24.03	0.828	0.440
	Fifth	41	60.23	24.13		
	Sixth	40	56.65	25.84		
Graphic creativity	Fourth	38	13.79	3.75	5.523	0.005^**^
	Fifth	41	11.38	4.24		
	Sixth	40	11.15	3.76		
General creativity	Fourth	38	66.72	25.72	0.359	0.699
	Fifth	41	71.60	25.26		
	Sixth	40	67.80	27.36		
Total test effectiveness	Fourth	38	227.58	50.05	13.658	0.000^***^
	Fifth	41	271.15	77.64		
	Sixth	40	303.05	59.98		
Intelligence	Fourth	38	99	12.55	8.658	0.000^**^
	Fifth	41	85.79	17.01		
	Sixth	40	88.96	17.15		

** p < 0.01;

*** *p < 0.001*.

ANOVA shows that no significant differences can be observed based on the educational level regarding narrative creativity (*F* = 0.828, *p* > 0.05) and general creativity (*F* = 0.359, *p* > 0.05). In graphic creativity, significant differences can be found regarding the educational level based on the results of ANOVA (*F* = 5.523, *p* < 0.01). *Post-hoc* tests results show that significant differences (*p* < 0.05) took place between fourth and fifth Elementary grades, with an average difference of 2.42. Other significant differences (*p* < 0.05) can be found between fourth and sixth Elementary grades, with an average difference of 2.65. ANOVA results (*F* = 13.658, *p* < 0.01) show significant differences regarding the total effectiveness of the test by educational level. *Post-hoc* data show that significant differences (*p* < 0.01) can be observed between fourth and fifth Elementary grades, with an average difference of −43.567. There are also significant differences (*p* < 0.01) between fourth and sixth Elementary grades, with an average difference of −75.471. ANOVA results (*F* = 8.658, *p* < 0.01) show that significant differences in IQ can be observed by educational level. *Post-hoc* data shows that significant differences (*p* < 0.01) were observed between fourth and fifth Elementary grades, with an average difference of 13.20. Significant differences can be also observed (*p* < 0.05) between fourth and sixth Elementary grades, with an average difference of 10.04, in students.

As previously stated, the concentration index is not evenly distributed. To address it, it was analyzed with a non-parametric method: Kruskal-Wallis *H*-test. Kruskal-Wallis test (*H* = 34.566, *p* < 0.01) shows that significant differences can be found in the concentration index by educational level. Significant differences (*p* < 0.01) were found between sixth (M = 85.85) and fourth (M = 43.49) Elementary grades. Significant differences (*p* < 0.01) can also be found between sixth (M = 85.85) and fifth (M = 50.09) Elementary grades.

The last goal was to determine if low attention levels positively affect creativity in students between the ages of nine and 12. As previously stated, Pearson correlation (Table 3) was used. Pearson correlation (*r* = 0.244, *p* < 0.01) shows that a relation between general creativity and total test effectiveness can be found, and that it is a low correlation since r's value is between 0.20 and 0.40. The same can be observed between narrative creativity and total test effectiveness, with a positive correlation, although with different results as shown in Pearson's correlation (*r* = 0.274, *p* < 0.01). The Pearson correlation for graphic creativity and total effectiveness (*r* = −0.100, *p* > 0.05), shows that no correlation exists between those variables.

**Table 3:** Correlations between creativity and attention.

**Variables**	**Statistics**	**General creativity**	**Narrative creativity**	**Graphic creativity**
Total test effectiveness	*r*	0.244	0.274	−0.100
	Sig.	0.007^**^	0.003^**^	0.277
	*N*	476	476	476
Concentration index	*r*	0.123	0.166	−0.212
	Sig.	0.182	0.072	0.021^*^
	*N*	476	476	476

* p < 0.05;

** *p < 0.01*.

Regarding possible correlations with the concentration index, the Pearson's correlation coefficient (*r* = −0.212, *p* < 0.05) for the concentration index and graphic creativity shows that a correlation between these two variables can be observed. This correlation is low, and at the same time negative, since r's value is between −0.20 and −0.40. This means that, due to a negative correlation, as the values of one variable diminish, the ones from another variable rise, and vice versa. Between general creativity and concentration index, results from Pearson's correlation (*r* =0.133, *p* > 0.05) shows that no correlation exists between these variables. In the same direction, results from Pearson's correlation (*r* = 0.166, *p* > 0.05) and between narrative creativity and concentration index show that no correlation among these variables exist.

With this last correlation comes the answer to the main question proposed by this research project. To confirm the hypothesis that low latent inhibition increases creativity, especially when a certain level of intelligence is present, the students selected for this research were those with an IQ higher that the average of this research, with the results listed on Table 4.

**Table 4:** Correlation between graphic creativity and concentration index in students an above-average IQ.

**Variable**	**Statistics**	**Concentration index**
Graphic creativity	R	−0.376
	P	0.003^**^
	N	240

** *p < 0.01*.

As reflected in Table 4, the negative correlation raises with the inclusion of the intelligence variable from the 240 participants' sample. The Pearson's correlation coefficient (*r* = −0.376, *p* < 0.01) decreases, raising when the negative significant correlation exists between the variables, although within the range of a low correlation.

## Discussion

At the start of this research, a set of goals and a hypothesis were proposed. The first specific goal was to get to know the creativity level, attention, and intelligence in students from nine to 12 years of age. Taking into account the results, creativity levels were diverse in each specific domain and in its general domain, due to the structure of the tests used, since three games evaluated narrative creativity, while only one evaluated graphic creativity (Artola et al., 2010). Regarding selective attention, levels of total effectiveness in the test are higher than the concentration index, as these last ones are lower, but more reliable since it penalized individuals who answer without control, taking into account correct answers, and thus showing a balance between stress and speed, avoiding overestimation (Brickenkamp, 2012). Regarding intelligence quotient (IQ), it shows low scores, since although a regular IQ pattern is shown, the average is 100, and thus almost eight points below the expectation (Yuste, 2011; Yuste et al., 2011). These IQ scores may be influenced by the context of the participants due to their low socio-economic background.

The second specific goal was to determine if differences in creativity levels, attention, and intelligence could be found based on gender. Data obtained for this research shows that gender is not a variable that determines creativity levels (Harris, 2004; Espinosa, 2005; Elisondo and Donolo, 2011; Abraham et al., 2014; Soisa, 2015). Gender is an irrelevant factor regarding selective attention (Fernández-Castillo and Gutiérrez, 2009; Brickenkamp, 2012; Soisa, 2015). The same findings were obtained regarding intelligence, since individuals between the ages of six to 14 do not show differences based on sex (Feingold, 1993; Lynn and Irwing, 2004).

Regarding the third specific goal, no significant differences could be found based on educational level regarding general creativity and narrative creativity. Regarding graphic creativity, significant differences can be observed in the higher levels of fourth grade. School lowers student's graphic creativity, since significantly lower scores can be observed in the fifth and sixth grades (Sternberg, 2007; Pérez, 2018).

For selective attention, significant differences on the educational level can be found based on the total effectiveness of the test, with the lowest levels observed in fourth grade students. Also, the concentration index shows significant differences based on the educational level, in which higher levels are shown on sixth grade students. Taking into account these results, older students show better scores in selective attention, since scores increase with age (Orjales and Polaino, 1992). This is due to the fact that, starting at about 10 years of age, students start to use selective strategies.

Regarding intelligence (IQ), data shows significant differences based on educational level, with the higher scores in fourth grade Elementary, showing higher IQ scores than the rest of the grade levels. Perhaps this is due to the data collection instruments, although the intelligence test scores were corrected for the age of the children.

It is confirmed, taking into account Pearson's correlation, that there is a relationship between concentration and graphic creativity variables. This is a negative correlation, since students with a lower selective attention present higher graphic creativity levels. Several works stated the idea that individuals showing less focus show higher levels of creativity (Campbell, 1960; Mednick, 1962; Mendelsohn and Griswold, 1966; Rawlings, 1985; González, 2017).

Regarding the research hypothesis, evidence was found that students with attenuated latent inhibition as the mechanism behind selective attention possess high graphic creativity, when a specific level of intelligence is present, as evidenced by above average IQ scores. This idea is supported by Eysenck's theory (1995) and numerous other research projects (Carson et al., 2003; Pritchard et al., 2006; González, 2017).

One of the main conclusions of this research is the concept that a creative individual is also a distracted individual, being frequently diagnosed only as inattentive and not creative (González, 2017), since from the educational point of view, it is easier to address inattentive students than creative ones. This labeling process can explain the higher number of ADHD students identified within the educational system, to the point of having to consider the possibility of over diagnosis. In educational practice, it is necessary to provide educational experiences appropriate to the characteristics of the creative and distracted child, developing in the classroom methodologies that allow for creative behavior and that are not so rigid as to allow moments of inattention as in the case of project-based learning.

Another conclusion is that creativity needs to be reconsidered within the educational system, in order to reinforce its implementation and development. Empirical evidence is provided showing that Eysenck's theory (1995), and research by Carson et al. (2003), confirms latent inhibition as the biological foundation of creativity, when the IQ requirement is met, although this work addresses the specific domain of graphic creativity. However, this association does not occur with the specific domain of narrative creativity.

## Data Availability Statement

The raw data supporting the conclusions of this article will be made available by the authors, without undue reservation.

## Ethics Statement

Written informed consent to participate in this study was provided by the participants' legal guardian/next of kin.

## Author Contributions

AL: data gathering, data analysis, and manuscript writing. OL-M: data gathering and manuscript writing. MV-Y: manuscript writing. All authors contributed to the article and approved the submitted version.

## Conflict of Interest

The authors declare that the research was conducted in the absence of any commercial or financial relationships that could be construed as a potential conflict of interest.

## References

[B1] Abraham A. Thybusch K. Pieritz K. Hermann C. (2014). Gender differences in creative thinking: behavioral and fMRI findings. Brain Imag. Behav. 8, 39–51. 10.1007/s11682-013-9241-4 23807175

[B2] Artola T. Ancillo I. Mosteiro P. Barraca J. (2010). PIC-N. Prueba de Imaginación Creativa para niños. Madrid: TEA Ediciones.

[B3] Ato M. López J. J. Benavente A. (2013). Un sistema de clasificación de los diseños de investigación en psicología. An. Psic. 29, 1038–1059. 10.6018/analesps.29.3.178511

[B4] Brickenkamp R. (2012). D2, Test de Atención (adaptación española de Seisdedos). Madrid: TEA Ediciones.

[B5] Campbell D. T. (1960). Blind variation and selective retentions in creative thought as in other knowledge processes. Psych. Rev. 67, 380–400. 10.1037/h0040373 13690223

[B6] Carson S. H. (2010). “Latent inhibition and creativity,” in Latent Inhibition: Cognition, Neuroscience and Applications to Schizophrenia, eds. Lubow B. Weimer I. (New York, NY: Cambridge University Press), 183–198. 10.1017/CBO9780511730184.010

[B7] Carson S. H. Peterson J. B. Higgins D. M. (2003). Decreased latent inhibition is associated with increased creative achievement in high-functioning individuals. J. Person. Social Psych. 85, 499–506. 10.1037/0022-3514.85.3.499 14498785

[B8] Chávez B. I. Ramírez J. Grimaldo E. (2020). Creatividad en la infancia: Diferencias por Edad y Sexo. REMAI 6, 34–46. Retrieved from: http://www.remai.ipn.mx/index.php/REMAI/article/view/69

[B9] Elisondo R. C. Donolo D. S. (2011). Los estímulos en un test de creatividad. Incidencias según género, edad y escolaridad. Bolet. Psic. 101, 51–65. Retrieved from: https://www.uv.es/seoane/boletin/previos/N101-4.pdf

[B10] Espinosa J. C. (2005). Incidencia del género y la edad en la creatividad infantil. Diver. Persp. Psic. 1, 22–30. 10.15332/s1794-9998.2005.0001.02

[B11] Eysenck H. J. (1995). Genius: The Natural History of Creativity. Cambridge: Cambridge University Press. 10.1017/CBO9780511752247

[B12] Feingold A. (1993). Cognitive gender differences: a developmental perspective. Sex Roles. J. Res. 29, 91–112. 10.1007/BF00289998

[B13] Fernández-Castillo A. Gutiérrez M. E. (2009). Atención selectiva, ansiedad, sintomatología depresiva y rendimiento académico en adolescentes. Elec. Jour. Res. Educ. Psych. 7, 49–76. 10.25115/ejrep.v7i17.1314

[B14] Gardner H. (2016). Estructuras de la mente: La Teoría de las Inteligencias Múltiples. Ciudad de México: Fondo de Cultura Económica.

[B15] Glaveanu V. P. Hanchett M. Baer J. Barbot B. Clapp E. P. Corazza G. E.. (2019). Avances en la teoría e investigación de la creatividad: un manifesto sociocultural. Uni-plur. 19, 97–106. 10.17533/udea.unipluri.19.1.07

[B16] González G. (2017). La creatividad en los niños con trastorno por déficit de atención con hiperactividad. [Dissertation thesis]. [Albacete (Spain)]: Universidad de Castilla-La Mancha.

[B17] Harris J. A. (2004). Measured intelligence, achievement, openness to experience and creativity. Person. Indiv. Differ. 36, 913–929. 10.1016/S0191-8869(03)00161-226161075

[B18] Hasher L. Lustig C. Zacks R. T. (2007). “Inhibitory mechanisms and the control of attention”, in Variation in working memory, eds. Conway A. Jarrold C. Kane M. Miyake A. Towse J. (New York, NY: Oxford University Press), 227–249. 10.1093/acprof:oso/9780195168648.003.0009

[B19] Hernández A. F. (2017). Un recurso de Innovación para docentes: Programa “Despierta Creatividad”. (dissertation thesis). Universidad de Murcia, Murcia, Spain.

[B20] Kaufman J. C. Baer J. (2004). Sure, I'm creative — but not in math!: self-reported creativityin diverse domains. Empir. Stud. Arts 22, 143–155. 10.2190/26HQ-VHE8-GTLN-BJJM

[B21] Kaufman S. B. (2009). Faith in intuition is associated with decreased latent inhibition in a sample of high-achieving adolescents. Psych. Aesthet. Creat. Arts 3, 28–34. 10.1037/a0014822

[B22] López O. Martín R. (2010). Estilos de pensamiento y creatividad. An. Psic. 26, 254–258. Retrieved from: https://revistas.um.es/analesps/article/view/109161

[B23] Lynn R. Irwing P. (2004). Sex differences on the progressive matrices: a meta-analysis. Intell. 32, 481–498. 10.1016/j.intell.2004.06.008 16248939

[B24] Martínez F. A. (2001). Creatividad: impulsividad, atención y arousal del rasgo al proceso. (Dissertation thesis). Universidad de Murcia, Murcia, Spain.

[B25] Mednick S. A. (1962). The associative basis of the creative process. Psych. Rev. 69, 220–232. 10.1037/h0048850 14472013

[B26] Mendelsohn G. A. Griswold B. B. (1966). Assessed creative potential, vocabulary level, and sex as predictors of the use of incidental cues in verbal problem solving. J. Person. Social Psych. 4, 423–431. 10.1037/h0023783 5969998

[B27] Orjales I. Polaino A. (1992). Déficit de atención selectiva y atención continua en niños con hiperactividad. Anál. Modif. Conduc. 18, 625–645. 3986035

[B28] Pérez L. (2018). “Imaginario, creación y creatividad,” in Perspectivas sobre la creatividad en educación, eds. Enríquez G. A. Pérez L. (Cuernavaca: Universidad Autónoma del Estado de Morelos), 13–27.

[B29] Pritchard V. Healey D. Neumann E. (2006). “Assessing selective attention abilities in ADHD, highly creative, and normal young children *via* Stroop interference and negative priming effects,” in Cognition and language: Perspectives from New Zealand, eds. Fletcher C. Haberman G. (Brisbane: Australian Academic Press), 207–224.

[B30] Rawlings D. (1985). Psychoticism, creativity and dichotic shadowing. Person. Indiv. Differ. 6, 737–742. 10.1016/0191-8869(85)90084-4

[B31] Runco M. A. (1994). Problem Finding, Problem Solving, and Creativity. New Jersey: Greenwood Publishing Group.

[B32] Sandoval C. (2016). La creatividad verbal como fortaleza pedagógica: diseño, aplicación y evaluación de un programa para Educación Primaria. (Dissertation thesis). Universidad de Murcia, Murcia, Spain.

[B33] Soisa J. (2015). Evaluación de la Creatividad, Atención e Inteligencia en alumnado de Educación Secundaria. (Master's thesis). Universidad de Extremadura, Cáceres, Spain.

[B34] Sternberg R. J. (2007). “Creativity as a Habit,” in Creativity: a handbook for teachers, ed. Tan A. G. (Singapur: World Scientific), 3–25. 10.1142/9789812770868_0001

[B35] Sternberg R. J. Lubart T. (1997). La creatividad en una cultura conformista. Un desafío a las masas. Barcelona: Paidós.

[B36] Taha V. A. Tej J. Sirkova M. (2015). Creative management techniques and methods as a part of the management education: analytical study on students' perceptions. Procedia 197, 1918–1925. 10.1016/j.sbspro.2015.07.563

[B37] Torrance E. P. (1977). Educación y capacidad creativa. Madrid: Ediciones Marova.

[B38] Valdivia M. R. (2018). “La medición y el muestreo,” in Metodología de la investigación cuantitativa-cualitativa y redacción de la tesis, eds. Ñaupas H. Valdivia M. R. Palacios J. J. Romero H. E. (Bogotá: Ediciones de la U), 323–345.

[B39] Yuste C. (2011). Batería de aptitudes diferenciales y generales renovado BADYG E2. Madrid: CEPE.

[B40] Yuste C. Martínez R. Galve J. L. (2011). Batería de aptitudes diferenciales y generales renovado BADYG E3. Madrid: CEPE.

